# Fatal Human Case of West Nile Disease, Mexico, 2009

**DOI:** 10.3201/eid1604.091614

**Published:** 2010-04

**Authors:** Clara Rios-Ibarra, Bradley J. Blitvich, Jose Farfan-Ale, Javier Ramos-Jimenez, Sissy Muro-Escobedo, Hector R. Martínez-Rodriguez, Rocío OrtizLópez, Ernesto Torres-López, Ana María Rivas-Estilla

**Affiliations:** Universidad Autónoma de Nuevo León, Monterrey, México (C. Rios-Ibarra, J. Ramos-Jimenez, S. Muro-Escobedo, H.R. Martínez-Rodriguez, R. OrtizLópez, E. Torres-López, A.M. Rivas-Estilla); Iowa State University, Ames, Iowa, USA (B.J. Blitvich); Universidad Autónoma de Yucatán, Mérida, México (J. Farfan-Ale)

**Keywords:** Viruses, West Nile virus, Mexico, human, neuroinvasive disease, letter

**To the Editor:** West Nile virus (WNV; family *Flaviviridae*, genus *Flavivirus*) was first recognized in the Western Hemisphere in 1999 during an outbreak of human, equine, and avian encephalitis in New York (*1*). The virus has since spread across the United States and Canada, where it has caused ≈30,000 human infections and ≈1,000 deaths. Serologic evidence has demonstrated that WNV is present throughout Mexico, Central America, South America, and the Caribbean region (*2**–**8*). However, WNV illness in humans and vertebrate animals in these regions has been only sparsely reported. For instance, 7 human cases of WNV infection have occurred in Mexico (excluding the case described here), 3 of which were severe. All patients survived. To our knowledge, no fatal human cases of WNV infection have occurred in Central America, South America, or the Caribbean region.

We describe a fatal case of WNV infection in a human in Central America. The patient, a man 40 years of age, lived in Monterrey, Nuevo León State, in northern Mexico. He had not traveled outside of the metropolitan area in the 6 months before illness onset. On June 11, 2009, influenza-like signs and symptoms (i.e., fever, malaise, fatigue, arthralgia, headache, and dizziness) developed in the patient. On June 26, the signs and symptoms had not resolved, and the man was admitted to University Hospital “Dr. José E. Gonzalez” at the Universidad Autonoma de Nuevo León (UANL). At the time of admission, cerebrospinal fluid (CSF) was collected, and laboratory analysis indicated a markedly elevated leukocyte count (182 cells/mm^3^; reference range 0–5 cells/mm^3^) and slightly elevated protein and glucose levels.

Several days later, serious neurologic signs that included loss of consciousness developed in the patient. On July 6, he lapsed into a coma and was transferred to the intensive care unit and treated for intracranial hypertension. Another CSF specimen was collected, and laboratory findings demonstrated that the leukocyte count had increased to 495 cells/mm^3^. CSF cytologic examination showed atypical lymphocytes, some of which resembled plasma cells. Brain magnetic resonance imaging showed hydrocephalus with no brain parenchymal lesions. Because the patient was suspected to have a herpes simplex virus infection, intravenous acyclovir was administered. Several days later, the patient showed signs of improvement; on July 15, he was discharged. Eleven days later, he experienced severe headaches and, on July 29, was readmitted to the UANL Hospital for intracranial hypertension. On July 30, a ventriculoperitoneal shunt was implanted; however, the patient’s condition continued to decline, and he died on August 1.

Personnel in the Laboratory of Molecular Infectology at the UANL were informed of the patient and were provided with the remainder of the second CSF specimen several days before his death. Total RNA and DNA were extracted from the CSF by using the QIAamp viral RNA extraction kit (QIAGEN, Valencia, CA, USA) and *DNAzol (Invitrogen,* Carlsbad, CA, USA) and tested for nucleic acid to various pathogens associated with human central nervous system infections, specifically herpes simplex virus types 1 and 2, human enterovirus A–D, dengue virus types 1–4, WNV, and *Mycobacterium tuberculosis*. Complementary DNA samples were generated by using Superscript III reverse transcription (Invitrogen), and PCRs were performed by using *Taq* polymerase (Invitrogen) in accordance with the manufacturer’s instructions. PCR amplifications were conducted by using the following reaction conditions: 94^o^C for 3 min; 30 cycles of 94^o^C for 1 min, 50^o^C for 1 min, and 72^o^C for 2 min; followed by a final extension at 72^o^C for 8 min. Reverse transcription–PCRs performed with diethyl pyrocarbonate–treated distilled water in place of nucleic acid were included as negative controls. All test and control reactions were performed in duplicate. PCR products were examined by 2% agarose gel electrophoresis and visualized with ethidium bromide. A PCR product of the expected size was observed when the WNV-specific primers WN-*cap*-F (5′-CAGTGCTGGATCGATGGAGAG-3′) and WN-*cap*-R (5′-CCGCCGATTGATAGCACT GGT-3′) were used. These primers amplify a 104-nt region of the capsid gene. All other assay results were negative. Subsequent reactions were performed by using a second set of WNV-specific primers, WN-*env*-F (5′-GATGTGAAGATGATGAATATGG-3′) and WN-*env*-R (5′-AATGCTTCCTTTGCCAAATAG-3′), which amplify a 216-nt region of the envelope gene. A PCR product of the expected size was again observed. PCR products were purified by using the Purelink Gel Extraction Kit (Invitrogen) and sequenced by using a 3730×1 DNA sequencer (Applied Biosystems, Foster City, CA, USA).

Because of the small volume of CSF obtained, a comprehensive laboratory analysis (virus isolation, plaque reduction neutralization test) could not be performed. Nevertheless, detection of WNV in the CSF of a patient with encephalitis meets the Centers for Disease Control and Prevention established criteria for a case of West Nile neuroinvasive disease (*9*). Our findings highlight the fact that the low number of WNV cases in Mexico and elsewhere in Latin America should not deter healthcare personnel from performing WNV diagnostic testing and the public from using personal protective measures in these regions.
